# Predicting Concentrations of Ultrafine Particles and Volatile Organic Compounds Resulting from Desktop 3D Printer Operation and the Impact of Potential Control Strategies

**DOI:** 10.1111/jiec.12578

**Published:** 2017-04-06

**Authors:** Parham Azimi, Torkan Fazli, Brent Stephens

**Affiliations:** Department of Civil, Architectural and Environmental Engineering, Illinois Institute of Technology, Alumni Memorial Hall Room 228, 3201 South Dearborn Street, 60616 Chicago, IL USA

**Keywords:** air emissions, air pollution, buildings, environmental health, exposure assessment, indoor air quality

## Abstract

Recent studies have shown that potentially hazardous volatile organic compounds (VOCs) and ultrafine particles (UFPs) are emitted from many desktop three-dimensional printer and filament combinations. We use recently published measurements of UFP and speciated VOC emission rates from a number of desktop 3D printers and filaments to predict the magnitudes of human exposures to airborne pollutants that would be expected in multiple locations within a typical small office environment. We also model the impacts of several control strategies for reducing occupational exposures. Results demonstrate that UFP and VOC concentrations within close or moderate proximity (i.e., within 3 and 3 to 18 meters, respectively) to some desktop 3D printer and filament combinations with the highest emissions can exceed recommended exposure levels (RELs) for some VOCs and typical indoor concentrations for both UFPs and VOCs. Concentrations of caprolactam within close proximity to a printer using some nylon-based filaments are predicted to exceed both acute and chronic RELs set by the California Office of Environmental Health Hazard Assessment. UFP concentrations are predicted to reach as high as 80,000 particles per cubic centimeter in close proximity to the highest emitting printer and filament combinations. The printer and filament combinations with the lowest UFP and VOC emission rates are not expected to yield concentrations at levels of concern. The most effective control strategies for reducing both UFP and VOC concentrations included installing a high-flow spot ventilation system and operating the printer in a sealed enclosure with high-efficiency gas and particle filtration.

## Introduction

Three-dimensional (3D) printers are rapidly gaining popularity for prototyping and manufacturing in a variety of industrial, educational, commercial, and residential settings. There are several additive manufacturing technologies used by 3D printers currently on the market (Afshar-Mohajer et al. 2015), although most relatively inexpensive desktop 3D printers utilize a technique called fused filament fabrication (FFF). In the FFF process, a 3D object is formed layer by layer as a thermoplastic filament is forced through a heated extrusion nozzle, melted, and deposited in thin layers onto a moving baseplate (Gross et al. 2014).

A few recent studies have shown that potentially hazardous gases and particles are emitted from many FFF 3D printers and filaments (Azimi et al. 2016; Kim et al. 2015; Steinle 2016; Stephens et al. 2013). Many printer and filament combinations have been shown to emit ultrafine particles (i.e., UFPs; particles smaller than 100 nanometers [nm]), whereas others have been shown to emit hazardous volatile organic compounds (VOCs) such as styrene and caprolactam (Azimi et al. 2016). Understanding the magnitude of these emissions has been critical, given that exposure to thermal decomposition products from acrylonitrile butadiene styrene (ABS) and other thermoplastics are known to have toxic effects in animals and humans (Oberdorster et al. 2005; Schaper et al. 1994; Unwin et al. 2013; Zitting and Savolainen 1980). Moreover, exposure to UFPs from a variety of sources have been linked with a variety of adverse health impacts in humans (Oberdörster 2000; Peters et al. 1997).

In a recent screening analysis, we estimated that some of the highest emitting printer and filament combinations that have been tested to date could yield high concentrations of VOCs and UFPs that could be of concern for human health when operated in small office spaces (Azimi et al. 2016). However, we are not aware of any studies to date that have explored in detail the likely human exposures to both gas and particle emissions from desktop FFF 3D printers and filaments in more realistic indoor environments. Therefore, in this work, we use recently published measurements of emission rates of both UFPs and speciated VOCs from a wide variety of desktop FFF 3D printers and filaments to predict the magnitudes of human exposures to these same pollutants that would be expected in multiple spatial locations within a typical small office environment. We also use the worst-case UFP and VOC emission scenarios to model the likely impacts of several potential control strategies for reducing exposures.

## Methods

### Characteristics of the Modeled Office Environment

**Figure 1:**
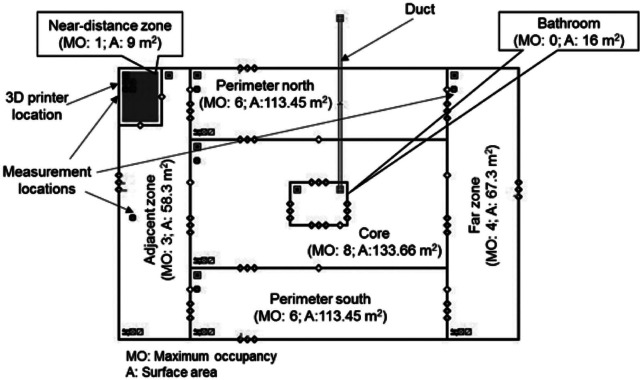
CONTAM model for the small office building. 3D = three-dimensional; MO = maximum occupancy; A = floor area; m^2^ = square meters.

We used a multizone airflow and contaminant transport analysis modeling software, CONTAM 3.2 (NIST 2012), to predict concentrations resulting from gas and particle emissions from several combinations of desktop FFF 3D printers and filaments, assuming each printer is operated inside a one-story office building with a floor area of 511 square meters (m^2^). The office building geometry and floor plan was taken from the Department of Energy Commercial Reference Building data set (Ng et al. 2012). However, we modified some of the building characteristics in the CONTAM model to represent more realistic scenarios. Figure 1 shows the building floor plan divided into multiple zones and the maximum occupancy in each zone (Ng et al. 2012).

In figure 1, each zone was assumed to be well mixed with uniform temperature, relative humidity, and pollutant concentration. In all simulations, one desktop 3D printer was located in the small shaded zone in the top left corner of figure 1. This zone does not have physical partitions like the other zones, but represents a 9-m^2^ floor area space within the western perimeter zone in which the printer is contained. We refer to this zone as the “near-distance” zone, in which a person would be sitting within immediate proximity to an operating 3D printer (i.e., within ∼3 meters [m]) for extended periods of time. We also refer to two other main zones in which occupants would be exposed to printer emissions only after the contaminants are transported from the printer location to those areas, including an immediately “adjacent zone” to the near-distance zone (within ∼3 to 18 m) and a “far zone” that is on the far end of the building (between ∼ 18 and ∼33 m away).

Each zone (other than the bathroom and the “near-distance” zone) was modeled with its own air-handling unit (AHU) designed to recirculate indoor air and deliver outdoor air for ventilation. The total supply and return airflow rates were the same for each AHU. We assumed that all five AHUs were designed to meet American Society of Heating, Refrigerating and Air-Conditioning Engineers (ASHRAE) Standard 62.1-2010 minimum ventilation requirements based on both floor area and occupancy (ASHRAE 2010). One exhaust fan ducted to the exterior was also assumed to operate continuously in the bathroom at an airflow rate of 1.8 cubic meters per hour (m^3^/hr). The required outdoor air ventilation rate for each zone, ${Q}_{vent,i}$ (m^3^/hr), was calculated using equation 1 (ASHRAE 2010):

1 $$ \kern0.33em {Q}_{vent,i}=\kern0.33em A\times {N}_{occupants,i}+B\times {A}_{zone,i} $$

where:

- *A* = the required outdoor air ventilation per person for a small office (9 m^3^/hr per person)
- *B*= the required outdoor air ventilation per floor area (1.08 m^3^/hr per m^2^)
- ${N}_{occupants,i}$ = the maximum number of occupants in zone *i* (-)
- ${A}_{zone,i}$ = the floor area of zone *i* (m^2^)

For the baseline simulation conditions, we also assumed that the AHUs met minimum heating, ventilation, and air conditioning (HVAC) filtration efficiency requirements in ASHRAE Standard 62.1-2010 with minimum efficiency reporting value (MERV) 8 particle filters and no gas-phase filtration (ASHRAE 2012). We estimated the removal efficiency of MERV 8 filters for UFPs emitted from desktop 3D printers to be 43.5% by mapping the size-resolved removal efficiency of filters recently tested in Fazli and Stephens (2012) to the resulting size-resolved distribution of emitted particles from 3D printers in an office environment reported in Stephens and colleagues (2013) for particles less than 100 nm following a procedure described in Azimi and colleagues (2014).

We assumed default building occupancy and HVAC system runtime schedules from the National Institute of Standards and Technology (NIST) model, with the building occupied on weekdays from 7:00 a.m. to 6:00 p.m. and the HVAC system operating from 6:00 a.m. to 10:00 p.m. on weekdays. In all cases, we assumed that a single desktop 3D printer operated continuously for 8 hours from 9:00 a.m. to 5:00 p.m., representing a relatively high, albeit not uncommon, duration of operation. We assumed that the building was located in Chicago, Illinois, and used Typical Meteorological Year (TMY) 3 data for Chicago (NSRDB 2012). Air infiltration through exterior walls was modeled using three characteristic openings in each wall representing various types of openings, including cracks and gaps in the walls, at the wall corners, and around large openings such as windows and doors. The effective leakage area was assumed to be 5.27 square centimeters (cm^2^)/m^2^ at a reference pressure difference of 4 pascals for all three types of small openings (Emmerich and Persily 2012). All of the zones were connected to at least one other zone by large openings with cross-sectional areas of 1 m^2^, which served to simulate doorway openings. The zone boundaries for the near-distance 3D printing zone were modeled as walls with large openings the same size as their cross-sectional areas (9 m^2^).

### Desktop Three-Dimensional Printer Emission Scenarios

Table 1 summarizes all of the recent measurements of particle and speciated VOC emission rates from desktop FFF 3D printers and filaments in the literature to date, including our recent study in which we measured both UFP and speciated VOC emissions tests for a total of five popular FFF 3D printers and nine different filaments, including ABS, polylactic acid (PLA), high-impact polystyrene (HIPS), semitransparent nylon, laybrick (an imitation brick material of unknown chemical composition), laywood (an imitation wood material of unknown chemical composition), transparent polycarbonate, a semitransparent nylon-based plasticized copolyamide thermoplastic elastomer (PCTPE), and a transparent polyester resin filament called T-Glase (Azimi et al. 2016). The three primary VOCs include caprolactam emitted from nylon-based filaments, styrene emitted from ABS and HIPS filaments, and lactide emitted from PLA filaments. For those pollutants that were consistently tested across all previous studies (i.e., UFPs from PLA and ABS), reported emission rates were generally within an order of magnitude of one another and thus the emission rates from Azimi and colleagues (2016) are considered reasonably representative of the other studies as well.

**Table 1:** Estimated particle and speciated VOCs from desktop 3D printers in existing literature

Reference	Filament	Particle emission rate (#/min)	Primary emitted VOC	VOC emission rate (μg/min)
Kim et al. (2015)	PLA	4.6 × 10^8^	Toluene	N/A
	ABS	1.6 × 10^10^	Ethylbenzene	N/A
Steinle (2016)	PLA	2.1 × 10^9^	Methyl methacrylate	6.5
	ABS	2.4 × 10^8^	Styrene	5.8
	PLA	1.1 × 10^8^	Lactide	4.4
	ABS	6.1 × 10^10^	Styrene	49.5
	HIPS	1.5 × 10^10^	Caprolactam	19.6
Azimi et al. (2016)	Nylon	2.4 × 10^9^	Caprolactam	182.6
	PCTPE	2.8 × 10^10^	Caprolactam	167.9
	Laybrick	6.9 × 10^7^	Caprolactam	69.1
	Laywood	9.8 × 10^7^	Caprolactam	45.4
	T-Glase	3.1 × 10^10^	Caprolactam	1.5
	Polycarbonate	6.2 × 10^10^	Caprolactam	1.5

Average of reported emissions in the referenced study.

VOCs = volatile organic compounds; 3D = three-dimensional; #/min = particles per minute; μg = micrograms; PLA = polylactic acid; ABS = acrylonitrile butadiene styrene; HIPS = high-impact polystyrene; PCTPE = plasticized copolyamide thermoplastic elastomer; N/A = not applicable.

Given the breadth of the Azimi et al. (2016) study, we use the emission rates of UFPs and the primary speciated VOCs emitted from each of the nine filaments to predict the time-varying concentrations of each pollutant that would likely be present in the previously defined near, adjacent, and far zones. We modeled the emitted pollutants “in addition” to what concentrations would have existed from other indoor sources. Thus, we assume that the concentrations of each pollutant in both outdoor and background indoor air are zero. In CONTAM, we assumed the diffusion coefficients of caprolactam and styrene in the office air are 0.065 and 0.071 cm^2^ per second (cm^2^/s) (NJDEP 2012), respectively, and kept the UFP and lactide diffusion coefficients equal to CONTAM defaults (0.2 cm^2^/s). We also assumed default CONTAM values for the effective density and specific heat of UFP equal to 1 grams per cm^3^ and 1 kilojoule per kilogram-kelvin, respectively. Finally, the UFP decay rate was assumed 25 × 10^−5^ s^−1^, equal to the median values measured in a recent study in UFP decay rates in residences (Wallace et al. 2013).

### Evaluating Exposure Control Strategies

We also modeled the impacts of several control strategies on resulting pollutant concentrations in the office space, including: (1) upgrading the central HVAC filtration throughout the office; (2) operating portable stand-alone air cleaners within close proximity to the 3D printer; (3) installing spot ventilation systems within close proximity to the 3D printer; and (4) operating the 3D printer inside a prototype sealed enclosure with recirculating gas and particle filtration. For this analysis, we modeled only the highest emission rates for each of the primary emitted pollutants to model the likely impacts of control strategies on only the worst-case scenarios (i.e., emission rates of 6.1 × 10^10^ particles per minute [#/min] for UFPs, 183 micrograms per minute [μg/min] for caprolactam, 50 μg/min for styrene, and 4 μg/min for lactide).

#### Upgraded Central Heating, Ventilation, and Air Conditioning System Filtration

In the first exposure control scenario, we assumed that the HVAC filters installed in the central AHUs were upgraded to MERV 16 media impregnated with activated carbon to provide both gas and particle filtration. We estimated the UFP removal efficiency of the MERV 16 filter to be 97% using the same matching approach with data from Fazli and Stephens (2012) described earlier for MERV 8 filters. Because there is a lack of literature on the gas-phase removal efficiency of impregnated activated carbon filters for caprolactam, styrene, or lactide, we relied on estimates from Sidheswaran and colleagues (2012) who reported VOC removal efficiencies of activated carbon fiber filters for six speciated VOCs ranging between 60% and 80%. Therefore, we assumed that the removal efficiency of the high-efficiency particulate arrestance (HEPA) filters with activated carbon fibers for all of the emitted VOCs is equal to 70%.

#### Operating Portable Stand-Alone Air Cleaners

Several previous studies have measured clean air delivery rates (CADRs) and/or single-pass removal efficiencies for UFPs from a number of commercial stand-alone air cleaners. Waring and colleagues (2008) reported a single-pass removal efficiency of ∼60% for two HEPA air cleaners with average CADRs across all particle sizes of 188 and 324 m^3^/hr, respectively. Other studies have reported CADRs for UFPs from other similar commercial air cleaner products ranging from ∼100 to ∼300 m^3^/hr, with single-pass efficiencies typically ranging from ∼45% to ∼60% (Mølgaard et al. 2014; Sultan et al. 2011). Therefore, we modeled two distinct portable air cleaner scenarios with CADRs of 100 and 300 m^3^/hr for UFPs. Assuming a single-pass efficiency of 60% for the HEPA filters, the airflow rates of the two air cleaners were calculated to be 167 and 500 m^3^/hr, respectively. We assumed that the filters also contained impregnated carbon with 70% removal efficiency for each of the VOCs modeled herein (Sidheswaran et al. 2012), which yields CADRs for the modeled VOCs of 117 and 350 m^3^/hr for the low- and high-efficiency air cleaner scenarios, respectively. Both air cleaners were modeled in CONTAM as an air handling system with equal supply and return air flows inside the near-distance zone and without any air exchange with outdoor air.

#### Spot Ventilation Systems

Next, we modeled the impacts of introducing spot ventilation in the near-distance zone within the immediate vicinity of the operating 3D printer. For lack of other data on exhaust systems that are designed specifically for 3D printer applications, we assumed that spot ventilation systems performed similar to exhaust hoods installed in small commercial or residential kitchens. We assumed that the hoods were located approximately 1.5 m above the desktop 3D printer and exhausted directly to the outdoors. A few recent studies in test kitchens have reported widely varying nominal and measured airflow rates, as well as capture efficiencies for both gas and particle emissions from cooking, that are achievable by a number of kitchen exhaust fans (Delp and Singer 2012; Logue 2012; Singer et al. 2012). The nominal airflow rates in these studies ranged from 325 to 1,300 m^3^/hr (with measured airflow rates from 75 to 650 m^3^/hr), and with low, moderate, and high pollutant capture efficiencies (CEs) ranging from less than 50% to greater than 75%, generally scaling with airflow rates (Singer et al., 2012). Moreover, if we were to consider the 67.3-m^2^ printing area in the office space as a “chemical storage room,” ASHRAE Standard 62.1 would require a minimum exhaust airflow rate of 27 m^3^/hr per m^2^ of floor area (i.e., a total exhaust rate of 1,817 m^3^/hr) to meet minimum ventilation requirements (ASHRAE 2010).

Given these bounds of reasonable airflow rates and CEs, we modeled three distinct spot ventilation scenarios with the following characteristics: (1) low effectiveness with 90 m^3^/hr (25 liters per second [L/s]) and 25% CE; 2 medium effectiveness with 360 m^3^/hr (100 L/s) and 63% CE; and (3) high effectiveness with 1,800 m^3^/hr (500 L/s) and 100% CE. To model the spot ventilation scenarios in CONTAM, we defined a hypothetical 1-m^2^ space inside the near-distance zone with an exhaust fan ducted to the outdoor air. We divided the emission sources between the zones based on the CE of the spot ventilation scenario. For example, if the CE was 25%, 75% of the total emissions of the four main pollutants were assumed to flow into the near-distance zone where they could be transported to other spaces and result in exposure, whereas 25% of the total emissions remained only in the 1-m^2^ “spot ventilation zone.” We divided the HVAC system supply airflow rates between the near-distance and spot ventilation zones in such a way that both zones have equal pollutant concentrations when the 3D printer operates and the spot ventilation airflow rate is zero.

#### Sealed Enclosure with Recirculating Filtration

Finally, we modeled the impacts of operating the 3D printers inside a prototype sealed enclosure with recirculating gas and particle filtration to reduce emissions. For this analysis, a pilot experimental chamber study was conducted with two high-emitting filament and printer combinations installed inside a prototype enclosure (Azimi et al. 2016). Average total VOC and UFP removal efficiencies of the enclosure were measured as ∼90% and ∼80%, respectively. For lack of other data, we assumed that the enclosure reduces the gas-phase emission rates equally (by 90%) for all speciated VOCs and all UFP emission rates equally (by 80%). The source emissions were simply reduced by 90% and 80% relative to the baseline scenario for gases and particles, respectively, in CONTAM.

## Results and Discussion

### Predicted Concentrations of Pollutants Emitted from Various Three-Dimensional Printer Filaments

**Figure 2:**
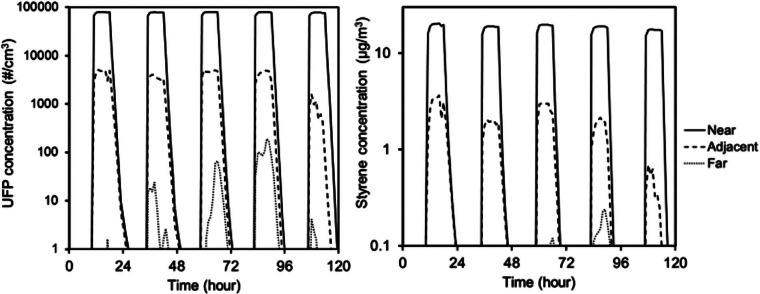
Typical hourly indoor concentration results from the CONTAM model with a single ABS printer operating in “near-distance,” “adjacent,” and “far” zones. UFP = ultrafine particle; #/cm^3^ = particles per cubic centimeter; μg/m^3^ = micrograms per cubic meter.

Figure 2 shows an example of modeled time-varying concentrations of UFPs and styrene in the three defined locations inside the small office environment during the weekdays of first week of January resulting from operating a single 3D printer with ABS filament (assuming the average ABS emission rate from data from Azimi and colleagues [2016] in table 1).

The modeled time-varying concentration results demonstrate that the peak exposure to pollutants emitted by the 3D printer in the near-distance zone are typically more than 9 and 4 times higher than in the adjacent zone for UFPs and styrene, respectively, whereas a much smaller fraction of pollutants would actually reach the far-distance zone. The model results also reveal that the increase in both gas and particle concentrations in the near and adjacent zones occurs almost immediately after the printer begins operating, whereas concentrations peak much later in the far-distance zone. Modeled concentrations in the far zone are also more variable because of changes in air infiltration conditions. For example, the relative standard deviation of the predicted maximum 1-hour concentrations throughout the entire year is only 2% for UFPs and 15% for styrene in the near zone, slightly more variable in the adjacent zone (41% for UFPs and 58% for VOCs) and most variable for the far zone (114% for UFPs and 106% for VOCs). These profiles are repeated with reasonable consistency for all weekdays throughout the year.

**Figure 3:**
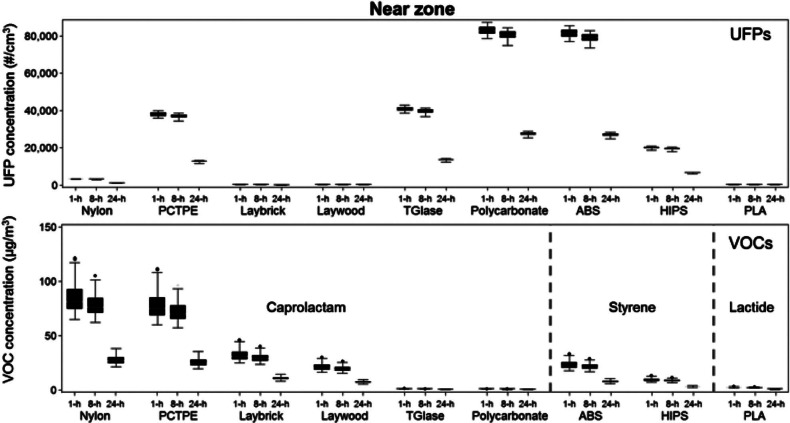
Ranges of predicted daily maximum 1-, 8-, and 24-hour concentrations of ultrafine particles (UFPs) and speciated volatile organic compounds (VOCs) in the “near zone” for a typical year assuming a single desktop 3D printer with nine different filaments operates continuously for 8 hours per day. ABS = acrylonitrile butadiene styrene; HIPS = high-impact polystyrene; PCTPE = plasticized copolyamide thermoplastic elastomer; PLA = polylactic acid; 3D = three-dimensional; #/cm^3^ = particles per cubic centimeter; μg/m^3^ = micrograms per cubic meter; 1-h = one hour concentration; 8-h = eight hour concentration; 24-h = 24 hour concentration.

**Figure 4:**
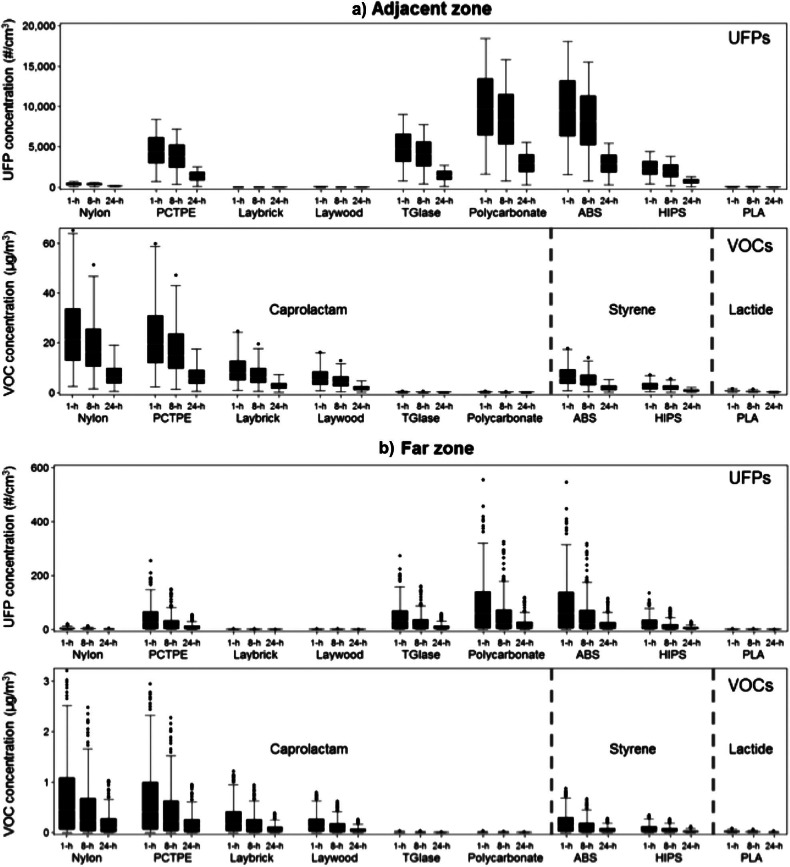
Ranges of predicted daily maximum 1-, 8-, and 24-hour concentrations of ultrafine particles (UFPs) and speciated volatile organic compounds (VOCs) in the (a) “adjacent zone” and (b) “far zone” for a typical year assuming a single desktop 3D printer operates with 9 different filaments continuously for 8 hours per day. ABS = acrylonitrile butadiene styrene; HIPS = high-impact polystyrene; PCTPE = plasticized copolyamide thermoplastic elastomer; PLA = polylactic acid; 3D = three-dimensional; #/cm^3^ = particles per cubic centimeter; μg/m^3^ = micrograms per cubic meter; 1-h = one hour concentration; 8-h = eight hour concentration; 24-h = 24 hour concentration.

Figures 3 and 4 summarize the predicted time-varying concentrations of UFPs and speciated VOCs in all three zones over the course of an entire year for each printer and filament combination. Figure 3 shows concentrations predicted in the nearest zone, and figure 4 shows concentrations predicted in both the adjacent and far zones. Results are presented as ranges of daily maximum 1-, 8-, and 24-hour concentrations (i.e., there is a total of 260 data points representing 260 weekdays in each series), because some of these metrics can be used to compare directly to regulatory limits. The daily maximum 8-hour concentrations during each weekday are calculated by taking 8-hour averages of the predicted hourly concentrations and selecting the highest average 8-hour concentration period for each day. The daily 24-hour concentrations are simply the daily averages for each weekday over the course of the entire year.

The median values of daily maximum 1-hour UFP concentrations in the near zone ranged from just under 100 particles per cubic centimeter (#/cm^3^) for Laybrick, Laywood, and PLA filaments to nearly 80,000 #/cm^3^ for ABS and polycarbonate filaments. Median values of daily maximum 1-hour VOC concentrations ranged from 0.7 to 80 μg/m^3^ with caprolactam-emitting filaments (highest with nylon and PCTPE), from 8 to 22 μg/m^3^ with styrene-emitting filaments (ABS and HIPS) and were consistently under 2 μg/m^3^ for lactide-emitting filaments. Because we assumed that the printer operates 8 hours per day, the maximum 1- and 8-hour daily concentrations are relatively similar, whereas the average 24-hour concentrations are substantially lower, with median values approximately 3% and 67% lower than the maximum 1-hour concentration, respectively.

The median values of daily maximum 1-hour UFP concentrations in the adjacent zone ranged from less than 20 #/cm^3^ for low-emitting filaments (e.g., Laybrick, Laywood, and PLA) to nearly 10,000 #/cm^3^ for ABS and polycarbonate filaments. The median values of daily maximum 1-hour VOC concentrations ranged from 0.2 to 20 μg/m^3^ with caprolactam-emitting filaments, 2 to 6 μg/m^3^ with styrene-emitting filaments, and were consistently lower than 1 μg/m^3^ for lactide-emitting filaments. UFP and VOC concentration followed similar patterns in the far zone, albeit at much lower absolute concentrations. The median 1-, 8-, and 24-hour UFP concentrations in the far zone were only 60, 20, and 7 #/cm^3^, respectively, when the 3D printer was modeled with polycarbonate and ABS filaments. The highest median 1-hour maximum concentrations of caprolactam, styrene, and lactide were only 0.5, 0.1, and 0.01 μg/m^3^ when the 3D printer was modeled with nylon, ABS, and PLA filaments, respectively. Thus, UFP and VOC emissions from the lowest-emitting filaments had essentially no impact on concentrations in the far-distance zone.

### Implications for Human Health

Predicted concentrations in the near and adjacent zones have important implications for human health. For example, the California Office of Environmental Health Hazard Assessment (OEHHA) maintains acute (i.e., 1-hour average), 8-hour average and chronic (i.e., continuous lifetime) reference exposure levels (RELs) of caprolactam of 50, 7, and 2.2 μg/m^3^, respectively (OEHHA 2012). Moreover, acute exposure to high concentrations of caprolactam is known to be “irritating to the eyes and the respiratory tract” and “may cause effects on the central nervous system” (CDC 2012). Results from the simulations herein suggest that operating a 3D printer with most of the nylon-based filaments (except T-Glase and polycarbonate) would increase the concentration of caprolactam in both the adjacent and near zones to levels that would exceed both the 8-hour and chronic RELs set by OEHHA. Further, the predicted caprolactam concentration in the near zone during printing with nylon and PCTPE filaments would also exceed the acute REL. The predicted caprolactam concentrations in the far-distance zone consistently remain under all RELs for all modeled nylon-based filaments.

Existing guidelines and recommendations for indoor styrene concentrations are limited to those set for industrial and workplace environments, including an 8-hour time-weighted average of 85 and 426 milligrams (mg)/m^3^ from the American Conference of Governmental Industrial Hygienists and the Occupational Safety and Health Administration Standard #29 CFR 1910.1000, respectively, and nearly 3,000 mg/m^3^ from the National Institute for Occupational Safety and Health. Further, the U.S. Environmental Protection Agency (US EPA) maintains a Reference Concentration for styrene of 1 mg/m^3^ based on central nervous system effects in occupationally exposed workers (US EPA 2012). However, other health impacts of styrene exposure have also been shown in epidemiology studies at much lower concentrations than these reference levels. For example, styrene concentrations as low as a few μg/m^3^ have been associated with elevated risk of pulmonary infections in infants (Diez et al. 2000). Our results demonstrate that the median styrene concentrations in the near-distance zone can easily reach more than 10 times this value and can be as much as 3 times higher than the highest measured styrene concentration of 7.5 μg/m^3^ in typical commercial buildings in the US EPA BASE study (Girman et al. 2012). Further, styrene is classified as a “possible human carcinogen” by the International Agency for Research on Cancer (IARC classification group 2B) (IARC 2012) and is “reasonably anticipated to be a human carcinogen” by the National Toxicology Program (National Toxicology Program 2014). These levels suggest that although the predicted styrene concentrations in all zones from all printer and filament combinations used herein are all lower than defined exposure limits, resulting concentrations still may pose a health risk to occupants.

Although we are not aware of any regulatory limits for indoor UFP concentrations, increases in UFP concentrations of ∼80,000 #/cm^3^ in the near-distance zone and ∼10,000 #/cm^3^ in the adjacent zone resulting from printing with polycarbonate and ABS filaments, as well as increases of ∼40,000 #/cm^3^ in the near-distance zone and ∼5,000 #/cm^3^ in the adjacent zone with some other filaments, are substantial, particularly given what is known about the health effects associated with outdoor UFPs. For example, UFP concentrations of ∼80,000 #/cm^3^ have been reported within 100 m of highly trafficked roadways (Zhu et al. 2002), and some of the observed associations of adverse health effects with proximity to busy roadways are likely attributable, in part, to elevated UFPs (McConnell et al. 2010; Gauderman et al. 2005, 2007). Further, recent studies have shown that increases in outdoor UFP concentrations of ∼10,000 #/cm^3^ are associated with a ∼3% increase risk in daily mortality (Stölzel et al. 2007), and increases in outdoor UFP concentrations of only ∼1,000 #/cm^3^ are associated with increased blood pressure in children (Pieters et al. 2015).

Comparing the modeled UFP concentrations to levels measured in other indoor environments, 80,000 #/cm^3^ is around 50% higher than the highest time-averaged UFP indoor concentrations that have been observed in schools in previous investigations (Diapouli et al. 2007). Printing with other filaments such as PCTPE, T-Glase, and HIPS would also increase the near-zone UFP concentrations to between 2 and 5 times higher than indoor UFP concentrations typically observed in other buildings in the adjacent and near-distance zones, respectively. Although much less is known about the adverse health effects of indoor-generated UFPs, recent studies of nanoparticles emitted from photocopiers and laser printers illustrate the potential hazard for human health. For example, UFPs collected from a university copy center were recently shown to induce lung injury and inflammation in mice (Pirela et al. 2013) and upper-airway inflammation and oxidative stress in healthy human volunteers (Khatri et al. 2013). These data suggest that controlling emissions and/or exposures from high-emitting 3D printer and filament combinations is warranted in settings similar to the one modeled herein.

### Impacts of Control Strategies on Pollutant Concentrations

**Figure 5:**
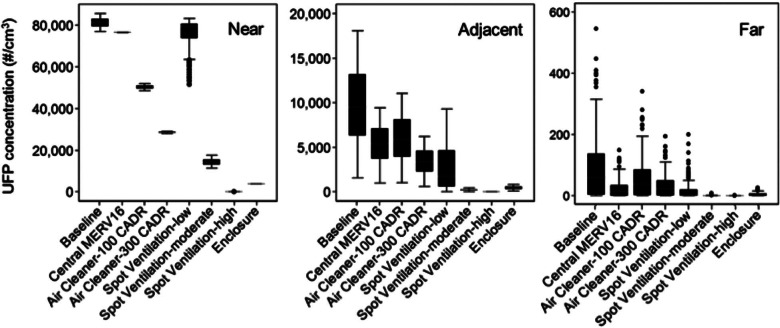
Modeled impacts of various control strategies on maximum 1-hour ultrafine particles (UFPs) concentrations in the near, adjacent, and far distances. CADR = clean air delivery rate; #/cm^3^ = particles per cubic centimeter; MERV = minimum efficiency reporting value.

Next, we chose the highest emitting printer and filament combinations for each pollutant and explored the impacts of various control strategies on the resulting concentrations in the same three locations within the modeled office environment. ABS, nylon, and PLA filaments were selected to represent the highest UFP and styrene, caprolactam, and lactide emissions, respectively. Figure 5 shows the predicted impacts of the various control strategies on maximum 1-hour UFP concentrations in the near, adjacent, and far distances with the 3D printer operating on the same schedule as all other simulations.

Upgrading the central HVAC system filter to a MERV 16 with activated carbon and installing a low-flow (and low capture efficiency) spot ventilation system are predicted to yield the smallest reductions in daily maximum 1-hour UFP concentrations in the near zone. Conversely, installing a high-flow (and high capture efficiency) spot ventilation system has the greatest potential for reducing UFP concentrations in the near zone, followed by using the sealed enclosure with particle and gas filtration. Both are predicted to reduce median values of daily maximum 1-hour UFP concentrations from ∼80,000 #/cm^3^ to less than ∼4000 #/cm^3^. Both air cleaner scenarios have moderate impacts on UFP concentrations in the near zone. These results suggest that in order to reduce exposures in areas within immediate proximity to operating 3D printers, it is best to prioritize solutions that exhaust or control emissions directly at the source rather than attempting to lower UFP concentrations in the broader area with air cleaners and filtration.

Results are somewhat similar in the adjacent zone, albeit with some variability. Installing a low-CADR portable air cleaner and upgrading the central HVAC system filtration are predicted to yield the smallest reductions in UFP concentrations in the adjacent zone, although the relative reductions in this zone are higher than those in the immediate vicinity of the printer. Both moderate- and high-flow spot ventilation systems, as well as the sealed enclosure, are predicted to yield the largest reductions in median values of daily maximum 1-hour UFP concentrations (all below 500 #/cm^3^). And although the air cleaners and low-flow spot ventilation system have lower UFP removal effectiveness, they can still limit increases in UFP concentrations to less than 5,000 #/cm^3^ in the adjacent zone. Similar relative concentration profiles are also observed for the far zone, but the baseline UFP concentrations are already quite low (∼60 #/cm^3^) and thus may not necessitate further control strategies.

**Figure 6:**
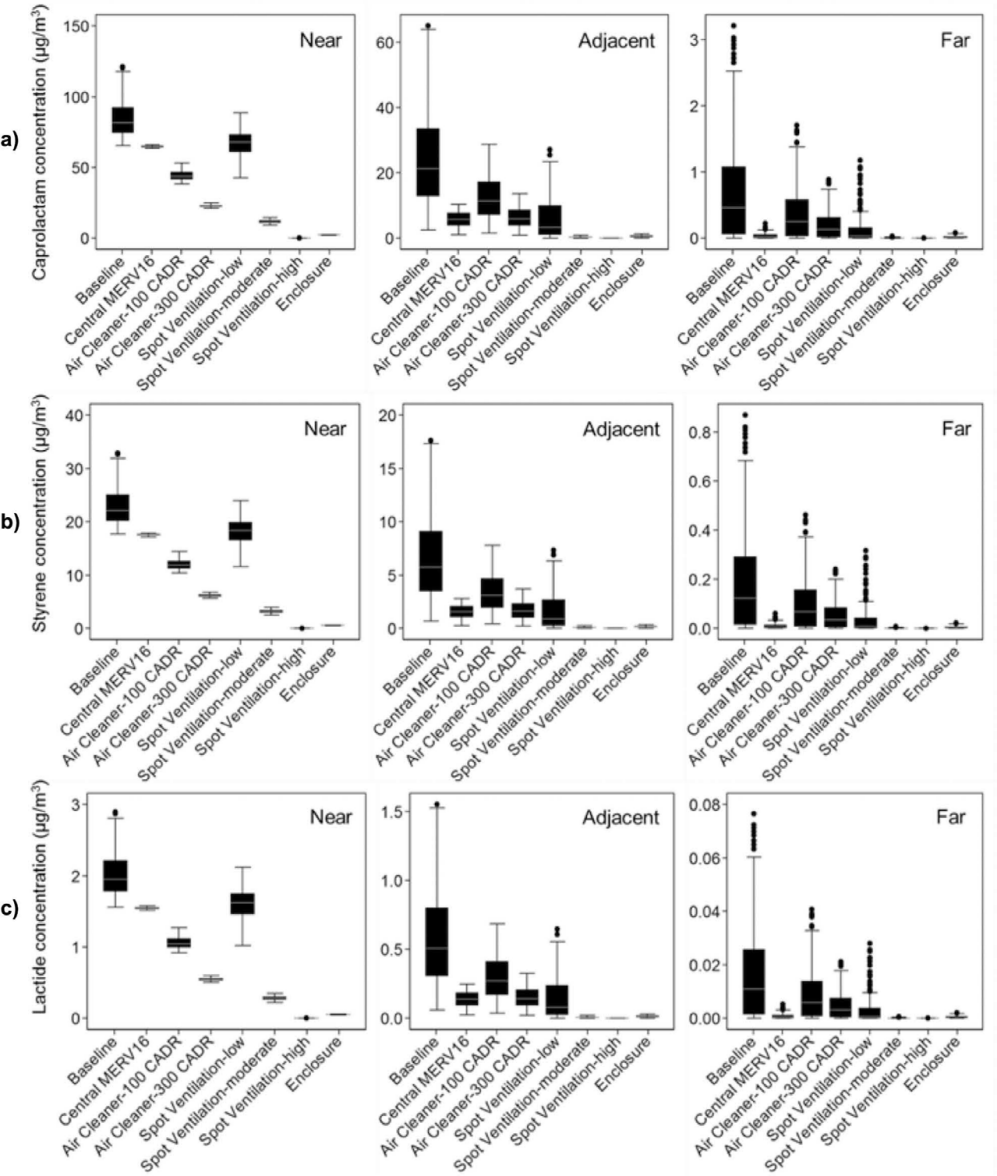
Modeled impacts of various control strategies on daily maximum 1-hour concentrations of (a) caprolactam, (b) styrene, and (c) lactide in the near, adjacent, and far zones. CADR = clean air delivery rate; μg/m^3^ = micrograms per cubic meter; MERV = minimum efficiency reporting value.

Figure 6 shows the impacts of the same control strategies for reducing daily maximum 1-hour concentrations of individual VOCs, including (1) caprolactam, (2) styrene, and (3) lactide emitted from nylon, ABS, and PLA filaments, respectively.

The relative reductions in daily maximum 1-hour concentrations with each control strategy are similar to those for UFP concentrations and are the same for all individual VOCs, because most of the same assumptions for pollutant dynamics apply to each VOC. However, absolute concentrations vary based on the strength of the emission source. In the near zone, upgrading central HVAC filtration and installing low-flow spot ventilation again have the smallest impacts on VOC concentrations, allowing most of the daily maximum 1-hour caprolactam concentrations from nylon-based filaments to still exceed the OEHHA acute REL of 50 μg/m^3^. The remaining control strategies would all reduce the median daily maximum 1-hour caprolactam concentration below this level. Both the high-flow spot ventilation and the sealed enclosure with gas and particle filtration are predicted to reduce the median daily maximum 1-hour caprolactam concentration to less than 2 μg/m^3^. Similarly, both moderate- and high-flow spot ventilation systems and the sealed enclosure are predicted to reduce the median daily maximum 1-hour styrene concentration to less than 7.5 μg/m^3^ (i.e., the maximum level measured in U.S. commercial buildings in the BASE study).

Installing moderate- and high-flow spot ventilation systems and the sealed enclosure are predicted to yield the largest reductions in VOC concentrations in the adjacent zone, reducing daily maximum 1-hour caprolactam and styrene concentrations to less than 0.5 μg/m^3^. The portable air cleaner scenarios yield smaller reductions in VOC concentrations in both the adjacent and far zones, but could still reduce peak caprolactam concentrations below the OEHHA acute REL for most days. All of the control strategies except for the low-CADR portable air cleaner are predicted to maintain the median daily maximum 1-hour styrene concentration in the adjacent zone below 2 μg/m^3^. Median daily maximum 1-hour lactide concentrations remain below 2 μg/m^3^ for all scenarios in all three zones, suggesting that lactide emissions from 3D printers using PLA filaments are likely not problematic for human exposure.

### Pollutant Removal Effectiveness of the Various Control Strategies

**Figure 7:**
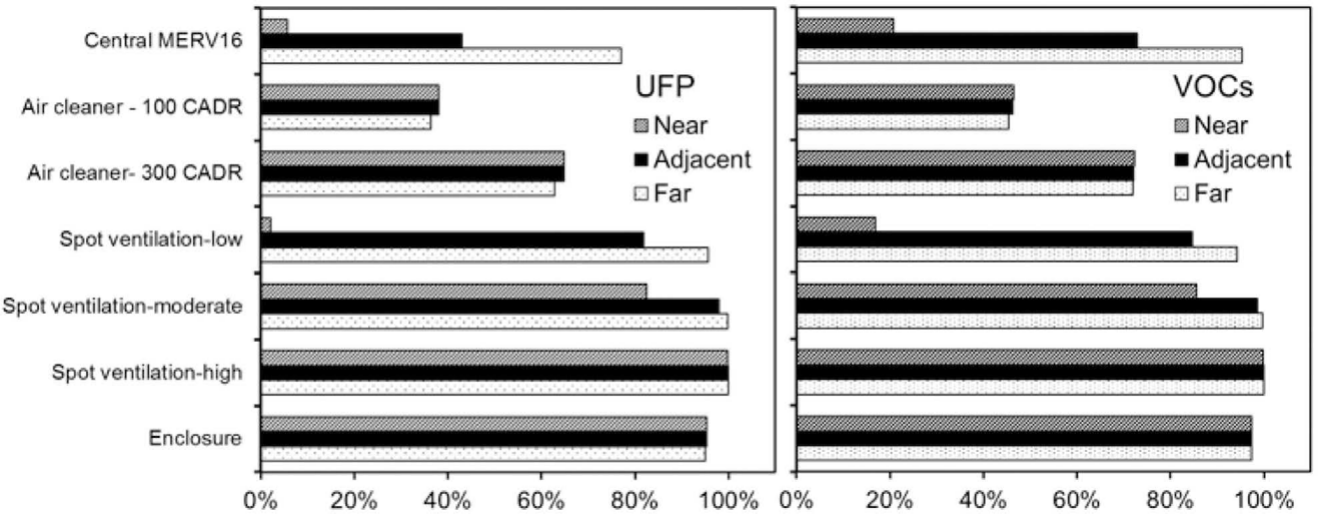
Removal effectiveness of each control strategy for reducing ultrafine particles (UFPs) and volatile organic compounds (VOCs) concentrations in the near, adjacent, and far zones. CADR = clean air delivery rate; MERV = minimum efficiency reporting value.

These same data were also used to calculate the effectiveness of each of the control strategies for reducing UFP and VOC concentrations in each zone using equation 2. Removal effectiveness values are shown in figure 7.

2 $$ \kern0.33em {E}_{control,i}=\kern0.33em 1-\frac{{\overline{C}}_i}{{\overline{C}}_{baseline,i}} $$

Where:

- ${E}_{control,i}$ = Effectiveness of a particular control strategy for removing UFPs/VOCs from a specific location (-)
- ${\overline{C}}_i$= Median concentration of UFPs/VOCs in a specific location predicted with the use of a particular control strategy (#/cm^3^ or μg/m^3^)
- ${\overline{C}}_{baseline,i}$ = Median concentration of UFPs/VOCs in a specific location predicted without the use of any control strategies (#/cm^3^ or μg/m^3^)

Removal effectiveness values range from as little as 2% for the expected impact of a low-flow spot ventilation system on UFPs in the near zone to between 95% and 100% for the expected impact of a high-flow spot ventilation system or sealed and filtered enclosure on both UFPs and VOCs in all zones. Both portable air cleaner scenarios have similar UFP and VOC removal effectiveness for all zones: 38% for UFPs and 46% for VOCs with the low-CADR air cleaner and 64% for UFPs and 72% for VOCs with the high-CADR air cleaner. The effectiveness of all other control strategies varied by zone. For example, the UFP and VOC removal effectiveness of upgraded central HVAC filtration is 6% and 21% in the near zone, 43% and 73% in the adjacent zone, and 77% and 95% in the far zone, respectively.

While these results provide insight into the likely impacts of realistic exposure control strategies on indoor concentrations of gas and particle emission products from desktop 3D printers, we should also note that some of these control strategies are more practical and cost-effective to apply than others. For example, high-flow rate spot ventilation systems that exhaust to the outdoors are likely cost prohibitive or impractical in many locations, and thus a sealed and filter enclosure may be a more appropriate solution in many environments. Further, operating a high-CADR stand-alone air cleaner with both gas and particle filtration can have a meaningful impact on both UFP and VOC concentrations in all zones, but could also come with a substantial energy penalty. We should also note that while the UFP and VOC emission rates from table 1 span a wide range of reasonable values, other printer and filament combinations will likely vary in both the magnitude of emissions and the types of individual VOCs emitted. Future work should also verify these findings with experimental data on both realistic exposures and the impact of control strategies in real environments.

## Conclusions

Results from the simulations herein provide insight into the likely magnitude of human exposures to gas and particle emission products generated by desktop 3D printers and provide practical recommendations for exposure control strategies. Modeled concentrations in the three zones demonstrate that UFP and VOC concentrations within close or moderate proximity to some operating desktop 3D printers can exceed recommended exposure levels and may be cause for concern for both acute and chronic health effects. The results also suggest that the most effective control strategies for reducing both UFP and VOC concentrations in all zones from high emitters used in the modeled environment, in descending order of impact, include: (1) installing a high-flow spot ventilation system; (2) operating the printer in a sealed enclosure with high efficiency gas and particle filtration; (3) installing a moderate-flow spot ventilation system; and (4) operating a high-CADR stand-alone air cleaner with both gas and particle filtration within immediate proximity to the operating 3D printer. Upgrading central HVAC filtration, installing low-flow spot ventilation, and operating a low-CADR portable air cleaner are the least effective methods for reducing UFP and VOC concentrations resulting from 3D printer operation in this modeled environment. Results also demonstrate that some 3D printer and filament combinations with lower emissions (e.g., PLA filaments, which have both low UFP and VOC emissions) should be prioritized over higher emitting filaments to limit human exposures.
